# The pandemic COVID-19: a tale of viremia, cellular oxidation and immune dysfunction

**DOI:** 10.11604/pamj.2020.36.188.23476

**Published:** 2020-07-15

**Authors:** Adekunle Babajide Rowaiye, Olukemi Adejoke Onuh, Angus Nnamdi Oli, Okiemute Ajiroghene Okpalefe, Solomon Oni, Ezinne Janefrances Nwankwo

**Affiliations:** 1 Department of Medical Biotechnology, National Biotechnology Development Agency, Abuja, Nigeria,; 2 Department of Pharmaceutical Microbiology and Biotechnology, Faculty of Pharm Scs, Nnamdi Azikiwe University, Awka, Nigeria,; 3 Genetics, Genomics and Bioinformatics Department, National Biotechnology Development Agency, Abuja, Nigeria,; 4 Bioresources Development Centre, Isanlu, National Biotechnology Development Agency, Abuja, Nigeria

**Keywords:** COVID-19, SARS-CoV-2, immune, viremia, cellular oxidation, immunoprophylactic, immunomodulatory

## Abstract

COVID-19, caused by SARS-CoV-2 is a tester of the immune system. While it spares the healthy, it brings severe morbidity and in a few cases, mortality to its victims. This article aims at critically reviewing the key virulence factors of COVID-19 which are the viremia, cellular oxidation and immune dysfunction. The averse economic effect of certain disease control measures such as national lock-downs and social distancing, though beneficial, makes them unsustainable. Worse still is the fact that wild animals and domestic pets are carriers of SARS-CoV-2 suggesting that the disease would take longer than expected to be eradicated globally. A better understanding of the pathological dynamics of COVID-19 would help the general populace to prepare for possible infection by the invisible enemy. While the world prospects for vaccines and therapeutic agents against the SARS-CoV-2, clinicians should also seek to modulate the immune system for optimum performance. Immunoprophylactic and immunomodulatory strategies are recommended for the different strata of stakeholders combating the pandemic with the hope that morbidities and mortalities associated with COVID-19 would be drastically reduced.

## Introduction

The current global pneumonia pandemic has been attributed to an outbreak of a novel coronavirus named Severe Acute Respiratory Syndrome Coronavirus 2 (SARS-CoV-2) which causes the infectious disease known as Coronavirus Disease 2019 (COVID-19) [1]. The first cases of this viral disease were recorded in December, 2019 in Wuhan, China [1]. As of April 15, 2020, a total of 1,914,916 confirmed cases with 123,010 deaths have been reported in about 212 countries [2]. SARS-CoV-2 is a novel β coronavirus [1] belonging to the coronaviridae family which are a group of enveloped, single-stranded, positive-sense RNA virus with a total genome size of 30kb [3]. SARS-CoV-2 is similar to other coronaviruses such as Severe Acute Respiratory Syndrome Coronavirus (SARS-CoV) and the Middle East Respiratory Syndrome Coronavirus (MERS-CoV) which are also zoonotic viruses responsible for outbreaks in 2003 and 2012, respectively [4]. SARS-CoV-2 may have originated from wild bats and was transmitted to humans [5]. Symptoms commonly seen in COVID-19 patients include fever, cough, sore throat, dyspnoea [1,4,5]. Occasional gastrointestinal tract symptoms like diarrhoea and vomiting may also occur [4]. Severe COVID-19 manifests as SARS, lung infections, end-stage renal disease and then, death [5,6]. Human to human transmission occurs via respiratory droplets with an incubation period of 1-14 days. The virus has also been detected in the faeces and urine of COVID-19 patients, suggesting a faecal-oral transmission route [1].

With a relatively low fatality rate, the immune system of victims plays a critical role in the survival of patients. COVID-19 is a tale of the immunopathology of SARS-CoV-2. Therefore, researchers and clinicians should not only look at inhibiting the virus or managing symptoms associated with the disease but also enhance the ability of the immune system both to prevent and combat COVID-19 [7]. Most viral pathogens cause acute, self-limiting infections due to an effective immune clearance. Morbidity and mortality in these infections is caused by the systemic imbalance caused by the immune system in the combat against the invading virus [8]. The major damage inflicted by COVID-19 is due to the overbearing effect of the immune system on virally infected cells. Therefore, the real killer is the exaggerated immune response to the virus [9]. Indeed, the immune system determines the severity of disease or virulence of pathogen. About 80% of COVID-19 patients manifest mild to moderate symptoms of the disease which disappear after about 6-10 days [2]. Fewer patients experience a cytokine storm and the complications of this uncontrolled inflammatory response are life-threatening. Even though the molecular dynamics underlying morbidity and mortality is yet to be fully understood, it is apparent that severe cases of COVID-19 are characterized mainly by an unbalanced immune response [9]. This objective of this case study is to present the pandemic COVID-19 with its associated viremia, cellular oxidation and immune dysfunction. It also offers greater orientation of the readers to immune education as regards the disease.

## Case study

### The pandemic COVID-19

**Figure 1:**
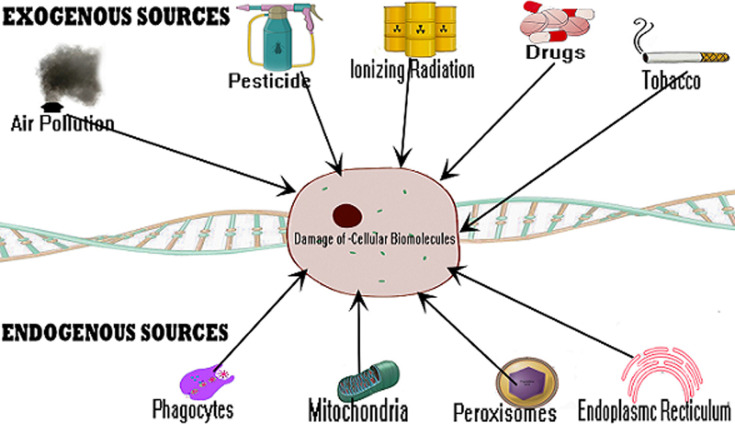
formation of free radicals

**Pre-infection: cellular oxidation and the immune system:** the immune system is an indicator of health and longevity and the cellular oxidation status is a marker of the strength of the immune system [10]. Biological systems generate free radicals as metabolic by-products during physiological, pharmacological and pathological processes [10,11]. Free radicals could be classified as reactive oxygen species (ROS) and reactive nitrogen species (RNS) and can be obtained from endogenous and exogenous sources. Endogenous sources include phagocytes, mitochondria, endoplasmic reticulum and peroxisomes while exogenous sources of free radicals include tobacco smoke, alcohol, ionizing radiation, pesticides, pollution, metals, organic solvents and drugs [11]. These are summarized in Figure 1. Free radicals play crucial roles in physiological processes like apoptosis, immunomodulation, cell signaling, ion transport and neuromodulation [12] and could be harmful thus required to be kept at very low levels [10]. Oxidative stress is a consequence of excessive generation of free radicals in cells which overwhelms the normal function of exogenous and endogenous antioxidants [10-12]. Due to their highly reactive nature, the accumulation of free radicals causes harmful effects on important cellular structures such as lipids, protein and DNA molecules [10]. This results in cytotoxicity or mutagenicity and form a fundamental part of many disease pathological processes [10,11]. On the contrary, ROS could have beneficial effects at physiological concentrations. For instance, phagocytes are known to produce ROS which are released to destroy pathogenic microbes when an invasion occurs [13].

Oxidative stress as a result of free radical damage to biomolecules is directly related to immune dysfunction and aging, while increased levels of antioxidants induces the improvement of immune function and longevity [14]. Oxidative stress has a direct relationship with inflammation; and oxidized molecules play a critical role in the regulation of immune cell function [15]. One of the functions of the mitochondria is ROS signaling which is important in the stimulation of the innate immune system through a biochemical cascade [16]. ROS are intracellular signaling molecules and when elevated, they are associated with immunosuppression. This is because ROS signaling induces T cell activation, apoptosis and weakened effector functions. Hence the effect of administering antioxidants combats T cells hypo-responsiveness [17]. Diet is the most common source of ROS generation. High sugar diet has been shown to cause oxidative stress through the production of ROS. The consumption of refined sugar significantly generates ROS through different pathways [18]. In the mitochondria, increased glucose metabolism produces reduced nicotinamide adenine dinucleotide and reduced flavin adenine dinucleotide. This increases superoxide generation which is converted to hydroxyl radical and hydrogen peroxide [18]. Thioredoxin - interacting protein which mediates high glucose-induced ROS production is upregulated by excessive glucose levels [19]. Insulin released due to high glucose levels results in hydrogen peroxide generation through activation of NOX4 [20]. Cardiovascular diseases such as cardiac arrhythmia, hypertension, atherosclerosis, cardiomyopathy and heart failure have also been linked to sugar induced-oxidative stress [18]. The World Health Organization advices that adults should reduce sugar and salt intake during the COVID-19 outbreak [21].

The major predilection site of COVID-19 are the lungs and excessive oxidative stress of the pneumocytes would make the lungs unfit to combat the disease. People living with lung cancers, asthma, chronic obstructive pulmonary disease (COPD), pulmonary fibrosis and acute respiratory distress syndrome (ARDS) are more likely to be overwhelmed with SARS-CoV-2 infections [22]. Tobacco smoking also increases oxidative stress of pneumocytes because tobacco smoke contains a plethora of harmful chemicals that generate lots of free radicals such as nitric oxide, nitrogen dioxide and peroxynitrite [12]. These radicals are released during peroxisomal metabolism, mitochondrial respiration and at the endoplasmic reticulum’s protein folding process with final stage ending in oxidative stress and pulmonary damage [23]. Remarkably, pulmonary neutrophilic inflammation is still evident in COPD patients even after tobacco smoking is halted [24]. Due to oxidative stress generated by Polycyclic Aromatic Hydrocarbons (PAHs), tobacco smoking enhances an increase in viral load in HIV infection and amplifies viral pathogenesis [25]. Individuals with stressed pulmonary system due to smoking, disease or pollution would be more vulnerable to the pneumotoxic effect of SARS-CoV-2 infection [26]. Drugs have been suggested to be a major exogenous source of ROS [27]. The mechanism by which drugs generate ROS and induce oxidative stress varies. A drug may generate a reactive intermediate which can transfer electron(s) to molecular oxygen, thereby creating ROS. For instance, doxorubicin an anthracycline antibiotic used in chemotherapy generates ROS when reduced by mitochondrial reductases to form anthracycline semiquinone free radicals [27].

These free radicals are unstable under aerobic conditions, thus reducing oxygen to super oxide anion and hydrogen peroxide [11]. Doxorubicin may also generate ROS by interacting with iron III through redox reactions to form iron II - doxorubicin free radical which has the ability to reduce molecular oxygen [27]. Oxidative stress caused by overwhelming levels of ROS generated by doxorubicin in cardiomyocytes could result in the activation of stress - linked pathways such as p38 and JNK, disruption of mitochondrial activities, accumulation of p53 and necrotic cell death [27]. Individuals that are constantly on drugs that weaken the immune system especially anticancer chemotherapeutic agents are more likely to have severe COVID-19 [28]. It has therefore been shown that antioxidants processes restore to normalcy blood pressure values in hypertension and also prevent some vascular alterations linked with aging [14]. Aerobic cells are equipped with antioxidant enzymes; superoxide dismutase (SOD), catalase (CAT) and glutathione peroxidase (GPx) that counteract the deleterious actions of ROS into less reactive or harmless molecules. There is correlation in individuals with oxidative stress/aged group and impairment of antioxidant defenses [14] as it results in abnormal levels of CAT, GPx and SOD activities in erythrocytes. As such, SOD, CAT and GPx are biomarkers of oxidative stress [14]. The coordinated antioxidant system counteracts the destructive effects of ROS. Antioxidants play an essential role in scavenging the reactive species and preserve an adequate function of immune cells [29]. Therefore, protection of the body’s defense system can be achieved by dietary antioxidant supplementation [30].

Specifically, antioxidants maintain the nature and function of membrane lipids, proteins and nucleic acids and as well control the signal transduction of gene expression in immune cells [30,31]. Improved antioxidant status enhances immunity [30]. Fruits, vegetables, vitamins (A, B, C, D and E) and other dietary supplements (such as selenium and zinc) contain antioxidants and would boost immune function against SARS-CoV-2 infection [31]. Immunocompromised individuals have a reduced ability to fight COVID-19 and other diseases [31,32]. Predisposing factors such as aging, poor nutrition, unhealthy lifestyle (like smoking, alcoholics) and underlining diseases such as diabetes mellitus, cancer and hypertension have been associated with immunodeficiency [33]. Mortalities in COVID-19 can be largely predicted by the number of comorbidities as 8 out of 10 deaths are occurring in individuals with at least one underlying disease [34]. Older adults are at high risk for severe illness from COVID-19 [35] and the elderly people are more prone to more serious outcomes when infected with SARS-CoV-2 especially acute respiratory distress syndrome (ARDS) and cytokine storm [36]. Over 95% of these deaths occurred in those older than 60 years and more than 50% of all deaths were people aged 80 years or older [34]. This may be as a result of immunosenescence which is an age-associated decline in immune function. All immune cells are affected by aging, thereby contributing to the high susceptibility to infection and increased mortality observed in the elderly [36]. Like other coronavirus infections and influenza, diabetes is a risk factor for severe COVID-19 disease. Mortality is three-fold higher in patients with diabetes than normal mortalities in COVID-19 [33].

This is due to the fact that diabetes is associated with an altered immune function. In diabetic patients, hyperglycemia impairs immune function by altering CD4 T cell activation. As compared to normal CD4 T cells primed by dendritic cells, these hyperglycemia-activated CD4 T cells have a reduced proliferation potential and a down-regulated expression of the apoptotic protein caspase-3 [37]. Therefore, a diabetic condition helps the spread of not only SARS-CoV-2 but also other secondary bacterial infection. Also, complications of diabetes make the management of COVID-19 more challenging [32]. Hypertension is a frequently occurring morbidity in the elderly and it is associated with immune dysfunction with the involvement of cells of the innate and adaptive immune systems. The production of proinflammatory cytokines such as TNF-α, IL-6 and IL-17 promote hypertension through the production of ROS, vasoconstriction and sodium reabsorption in the kidney [38]. Individuals with this condition are at risk of having severe forms and complications of COVID-19 [39]. Among older patients, hypertension is associated with a 2.5-fold higher risk of severe disease and mortality [40]. However, renin-angiotensin system inhibitors have been known to improve clinical outcomes [41]. Cardiovascular disease (CVD) is an immune-related pathology. Thrombosis formed by the aggregation of inflammatory cells is found at sites of arteriosclerotic plaque disruption. Furthermore, T lymphocytes and macrophages secrete cytokines, growth factors and metalloproteinases which stimulate smooth-muscle growth, extracellular matrix secretion and alter the general architecture of the plaques [42]. Emerging data suggests that patients with preexisting CVD are at a high risk of mortality from COVID-19 [4,42]. Co-morbidity with CVD was reported to have increased mortality especially in the older people [4]. COVID-19 also causes cardiovascular complications such as myocarditis, arrhythmias, acute heart failure, acute coronary syndromes, cardiogenic shock and death [42].

### Infection with SARS-CoV-2

**Figure 2:**
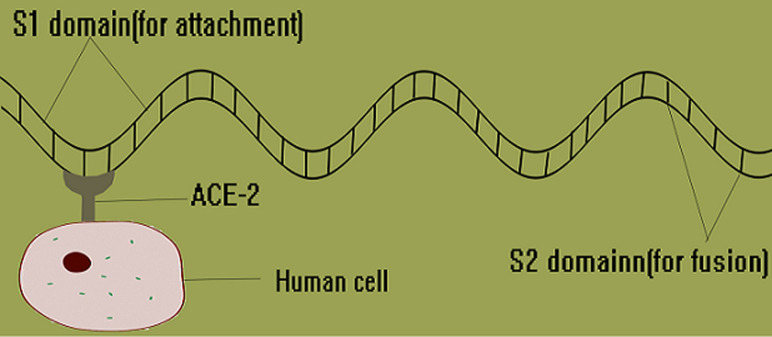
SARS-CoV-2 spike protein attaching to human cell

**Viral pathogenesis:** like other viruses, SARS-CoV-2 multiplies through several processes such as attachment and penetration of host cell wall; uncoating and replication within the host cell; assembly of viral particles and release outside the host cell [43]. Attachment and fusion to host cell is through the spike protein of the SARS-CoV-2 (Figure 2). The spike protein has S1 and S2 domains domiciled at the N and C termini respectively [43]. The S1 domain is used for attachment to the Angiotensin Converting Enzyme 2 (ACE2) through the receptor-binding domain (RBD) while the S2 domain contains a putative fusion peptide and two heptad repeat regions (HR1 and HR2) and it is responsible for fusion to the host cell membrane through mediate membrane fusion in both pH-dependent and -independent endocytosis [43,44]. ACE2 is a cell surface metallopeptidase expressed on human pneumocytes (types I and II), enterocytes of the small intestine, kidney proximal tubule brush border cells, endothelial cells of arteries and veins, and smooth muscle cells in several organs [44]. However, the major predilection site of SARS-CoV-2 is the lungs and upper respiratory tract. ACE2 blocks the activity of Angiotensin Converting Enzyme (ACE) by converting angiotensin II (a vasoconstrictor peptide) into angiotensin (1-7) (a vasodilator) thereby lowering the blood pressure and plays both cardio and pnuemo-protective roles [45]. The inactivation of ACE2 by blocking with the spike protein worsens pulmonary hemodynamics culminating in acute lung injury (ALI) and also causes a deterioration of cardiac activity [44,45]. Therefore, direct ALI is caused by the virus damaging the bronchial and alveolar epithelium and indirect pulmonary injury by immune-mediated responses.

**Figure 3:**
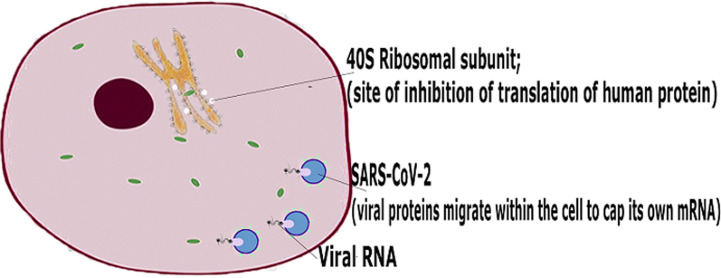
SARS-CoV-2 pathogenesis

After invasion of host cell, the SARS-CoV-2 releases its RNA genome into the cytoplasm of the host cell and hijacks the replication process. Unlike HIV which integrates its genome into the nucleus, through the help of the protease, reverse transcriptase and integrase enzymes, SARS-CoV-2 remains in the cytoplasm [46]. The nonstructural protein (NSP) 1 of SARS-CoV-2 binds to the 40s ribosomes of the host cell to inhibit its native function of translating proteins. Furthermore, this ribosomal binding induces the degradation of the mRNA of the host cell through endonucleolytic RNA cleavage near the 5’UTR end. The viral mRNA is spared of this cleavage because of the methylation and capping executed by a viral enzyme, Guanine-N7 methyltransferase [46,47]. Within the host cytoplasm, the RNA genome of the virus is then translated into structural and non-structural proteins, assembled and released outside the cell (Figure 3). The structural proteins which include the spike (S) glycoprotein, membrane (M) glycoprotein, envelope (E) glycoprotein and nucleocapsid (N) protein are responsible for the formation of the viral architecture [48]. The non-structural proteins (NSP) are responsible for vital physiological processes such as RNA synthesis, replication, assembly and release. Some NSPs which are potential drug targets include single-stranded RNA-binding protein and enzymes such as main protease, papain like protease, helicase, RNA-dependent RNA polymerase, guanine-N7 methyltransferase, 2' O-methyltransferase, ADP-ribose-1”-phosphate phosphatase, primase, 3'5' exoribonuclease and poly (U)-specific endoribonuclease [46].

**Immune evasion:** as with other viruses, SARS-CoV-2 possesses innate mechanisms it deploys in maintaining molecular anonymity in a bid to evade the immune system as evidenced in the delay of virally infected cells to release type I interferons (IFN alpha and beta) [49]. As found in SARS, there is a suppression of the release of interferons by infected cells which delays immune response in COVID-19 [7]. Specifically, the SARS-CoV-2 also evades immune attack by an mRNA capping mechanism facilitated by guanine-N7 methyltransferase [49]. SARS-CoV-2 also attacks the lymphocytes causing lymphocytopenia which is a common diagnostic marker in COVID-19 patients [50]. Though ACE2 is not found on T and B cells, SARS-CoV-2 invades T-lymphocytes through the CD147 molecule [50]. Similar to the histopathological condition found in SARS and MERS, SARS-CoV-2 does not replicate in T-lymphocytes but rather renders them ineffective [51].

**Viremia and oxidative stress:** oxidative stress is a mechanism of pathogenesis observed in RNA viruses. Viruses induce oxidative stress by activating the production and release of ROS by phagocytes (oxidative burst); at the same time cause depletion in antioxidant defense systems [52]. This creates a situation whereby the body is unable to balance the production and detoxification of ROS, consequently generating oxidative stress. Activated phagocytes release not only ROS but also proinflammatory cytokines. In the presence of oxidative stress, viral replication is activated due to the presence of nuclear transcription factor (NF-κB) [52]. While immune cells generate ROS when fighting against infections, they also utilize ROS in support of their functions to kill pathogens through immune activation; though the process also may cause oxidative damage to host tissues [53]. Therefore, supplementary dietary antioxidants are needed in the management of COVID-19 to improve immunity [30]. The aggressive administration of exogenous antioxidants from natural products would largely impart on clinical outcomes of COVID-19 by improving patient’s health, hastening the immunological process and consequently shortening the hospital admission period.

### Immune response to SARS-CoV-2 infection

**Innate immune response:** the innate immune system serves as the first line of defense in the combat against the viremia associated with COVID-19. Studies carried out on the SARS-CoV-2 suggest that like SARS-CoV, a major causative factor of morbidity and mortality in humans is the dysfunctional innate immune response [36]. At the site of infection, damaged cells elicit innate response by mobilizing pro-inflammatory granulocytes and monocytes from the blood and the lymph. Interestingly, the role of NK cells in SARS infections has not been established but they have been reported to be reduced in peripheral blood in patients with severe cases of COVID-19 [50]. Usually a faulty frontline defense could be due to factors such as overwhelming number of invaders, the number and activity of the immune cells responding. The monocytes (dendritic cells and macrophages) at the site of infection upregulate the expression of pro-inflammatory cytokines and chemokines such as RANTES, Macrophage Inflammatory Protein (MIP)-1α, and Monocyte Chemoattractant Protein (MCP)-1, CD14, TLR9, CC chemokines (CCL4, CCL20, CCL22, CCL25, CCL27, IL-1, IL-2, IL-4, IL-6, IL-8 and IL-17) [53]. Also elevated in lungs and peripheral blood include IFN-γ (type II interferon), IFN-inducible protein-10 (IP-10) and the monokine induced by interferon-gamma (Mig). The level of anti-inflammatory cytokines such as TGF-β levels was not increased in patients [53]. Interestingly, type 1 IFN is not detected in COVID-19 patients and also not detected in *in vitro* SARS-CoV-2 infected cells [54]. The administration of type 1 IFN to COVID-19 would trigger interferon-mediated immune responses which could have a positive impact on the clinical outcome of severe cases [55].

**Adaptive immune response:** dendritic cells derived from monocytes trigger the adaptive immune system by presenting the viral antigen to the T and B cells. While T cells especially the CD8 help destroy virally-infected cells, B cells which produce antibodies prevent new cells from being infected. The CD8 cells recognize viral peptides presented on the surface of the infected cells and elicit cytotoxic activity. Epitopes from S, E and M proteins elicit cellular immune responses as revealed by an upregulation of interferon-γ and interleukin-4 production [50]. Notably, the activation of the CD8 cells is the major cell-mediated immune response against SARS-CoV-2. This is because as it is with SARS-CoV infection, the acute phase of COVID-19 is characterized by a remarkable reduction in CD4 T cell population and this could be prolonged reaching a lowest point at days 7-9 before returning to normal [50].

**Humoral response:** neutralizing antibodies which specifically incapacitate the spike glycoproteins are produced by B cells and prevent the attachment of the virus and completely protect the host [50]. In a 47-year-old female patient with mild case of COVID-19, antibodies were detected as early as four days after infection while the virus could be undetected as early as the 7^th^ day and there were progressive increases in plasma SARS-CoV-2-binding IgM and IgG [56]. While the antibodies for SARS and MERS may last for over a year, the duration of that of SARS-CoV-2 is yet to be determined. However, scientists infected the rhesus monkeys with SARS-CoV-2 resulting in a mild form of the disease. The monkeys developed neutralizing antibodies within a few days after infection, recovered and did not show the symptoms of disease when re-infected [57]. Though cases of reinfection have been reported in South Korea, these might likely be a relapse of the disease [58].

**Virus-activated cytokine storm:** when both innate and acquired innate immunity fails, there is a protracted and irrational mobilization of immune cells to the site of infection. These cells in turn release a gust of proinflammatory cytokines which damage healthy tissues [59]. CD8 cells attack the virally infected cells of the thin alveolar epithelium. Cytotoxic cytokines from these cells also destroy the alveolar capillary endothelium causing protein rich fluid to leak into the alveolar space. This uncontrolled inflammatory response damages alveolar tissue; impairs oxygen transportation in the lungs and causes Acute Respiratory Disease Syndrome (ARDS) [59]. The cytokine storm is a potentially harmful amplification of inflammatory response which is contrary to the physiology of the pulmonary system. For example, alveolar macrophages are poor antigen presenting cells. Therefore, to avoid ARDS, it is important that the lungs continue to maintain a blunt immune response as this ensures optimal function [7,59]. The pathophysiological concept of cytokine storm has it underlying genetic signatures. Individual genetic polymorphism is also a major determinant of individual immune responses and also ultimately the severity of the disease [7]. Human Leucocyte Antigen (HLA) molecules belonging to the MHC 1 complex determine susceptibility to viral diseases [60]. The inability of HLA molecules to present viral antigenic peptides to the surface of infected cells would culminate in poor immune responses. This might be due to mutations in the HLA gene complex. Viral diseases such as Lassa fever, H1N1 and ebola infections recognize different HLA molecules [61]. Individuals with HLA-B*46: 01 are reported to be more vulnerable to COVID-19 [53]. The discovery of the specific HLA molecules and variants involved in COVID-19 would enhance knowledge on the unexplained susceptibility to the disease.

It is more ideal to talk about a balanced immune system rather than a strong one. The degree of lymphopenia is more pronounced in a cytokine storm than in mild or moderate cases [62]. Unlike in mild cases of COVID-19, severe cases would have an unusually high level of pro-inflammatory cytokines and chemokines such as IL-6, IL-8, MIP-1β, MCP-1 and IFN-γ6, in the plasma. Lymphopenia (in CD4+ and CD8+ T cells) and decreased IFN-γ expression in CD4+ T cells are also associated with severe COVID-19 [9]. This virus-induced cytokine storm is capable of causing heart failure, acute renal failure, unresolved diarrhea, respiratory failure and neurological failure [56]. To enhance a more effective management of COVID-19, the early prediction of severe cases or high-risk individuals is important. Of all cytokine profiles and the cell counts, the Neutrophil to Lymphocyte Ratio (NLR) is the most powerful prognostic indicator in the identification of severe cases of COVID-19 [9,62]. With this surrogate marker, the early prediction of a cytokine storm is possible. The NLR can easily differentiate between severe and non-severe cases and also predict their clinical outcomes [63]. Severe cases usually have higher NLR. In a study of 245 COVID-19 patients admitted in hospital, NLR of patients in the highest interquartile had 15.04-fold higher risk of death than patients in the lowest interquartile [62]. Even in AIV-H7N9 influenza viral infections and ARDS, NLR remains a predictive prognostic biomarker of clinical outcomes in ill patients [64]. Cytokine storm can be controlled by blocking pro-inflammatory cytokines. Specifically, blocking IL-6, IL-1 and TNF may be beneficial to patients [7]. Anti-inflammatory mediators such as cyclo-oxygenase1 can also be activated. Inhibition of HA-synthase-2 (HAS2) which is an enzyme induced by pro-inflammatory cytokines (IL-1 1β, TNFα) is also another therapeutic strategy. 4-methylumbelliferone is a known HAS-2 inhibitor [65]. Hyaluronidase can also be administered intranasally [7] and computational approaches present a potential drug discovery option for the COVID-19 [66].

**Chloroquine, a double agent:** chloroquine (CQ) and its derivative hydroxychloroquine (HCQ) have been reported to show good promise in the treatment of COVID-19 this is because they present a dual advantage of quelling the cytokine storm and inhibiting the coronavirus [67]. CQ has been used to shorten the course of the disease and it has been enlisted as one of the trial drugs in the guidelines for the diagnosis and treatment of COVID-19 published by national health commission of China [68]. In many countries, clinical trials have been approved to validate the efficacy and to establish the ideal therapeutic dose for the treatment of COVID-19. Of these ongoing trials (as at May, 2020), five out of seven completed ones reveal that the use of CQ or HCQ in patients had favourable clinical outcomes. However, there are concerns about varying degrees of bias and poor design [69]. CQ is a readily available, cheap antimalarial but an overdose usually leads to acute poisoning and death [70]. Therefore, HCQ, which is a less toxic derivative, is preferred. HCQ and CQ exhibits immunomodulatory activity and has been used in the treatment of auto-immune diseases such as antiphospholipid syndrome, rheumatoid arthritis, systemic lupus erythematous, primary Sjögren syndrome and other inflammatory rheumatic diseases [68]. Being weak bases, CQ and HCQ accumulate in the acidic intracytoplasmic vacuoles such as lysosome, endosomes and golgi apparatus.

This alters the pH of these vacuoles disrupting physiological processes such as protein degradation by acidic hydrolases, assembly of macromolecules and post translational modification of proteins respectively [68]. At the cellular level, CQ and HCQ inhibit immune activation directly or indirectly by depressing leucocyte function. Being weak bases, they increase the pH of cytoplasmic compartment and this disrupts antigenic protein digestion and assembly in at the alpha and beta chains of MHC class II proteins of macrophages and other antigen-presenting cells [68,70]. Due to a diminished formation of peptide-MHC protein complexes, the stimulation of CD4+ T cells is disrupted and consequently, there is reduced toll-like receptor signaling, reduced cytokine production by T cells and downregulation of the expression of CD154 which is a co-stimulatory molecule [68]. CQ and HCQ disrupt antigen-antibody reactions through the inhibition of complement activity [68,69]. Though these agents act like glucocorticoids in their anti-inflammatory and immunosuppressant activity, they have a slow onset of action but altogether, these properties make CQ and HCQ good candidates in combating cytokine storms. With a similar mechanism, HCQ and CQ exhibit antiviral properties by increasing the pH of intracytoplasmic organelles thereby leading to the unfolding of proteins of invading viruses. Another antiviral mechanism of CQ is the disruption of the terminal glycosylation of the cellular receptor ACE2 which prevents binding of SARS-CoV [66,68].

**Recommendations:** vaccine and immune development: the race for the development of a vaccine against COVID-19 is on with the first clinical trial presently taking place in the UK. Bearing in mind that there were outbreaks of SARS and MERS in 2003 and 2012 respectively, it is logical to assume that there would be more outbreaks of mutated strains of the coronavirus in the future. To sustain the efficacy of prospective vaccines, it is important to also use immunogenic peptides from conserved regions within the virus. Emerging data suggests that pets are potential reservoirs of SARS-CoV-2, therefore animal vaccines and antivirals should also be developed. An extensive serological survey is also required to determine the antibody titre of post-COVID-19 patients. This would help for a better understanding of the immunological memory built by the body against SARS-CoV-2 as compared with other species of coronaviruses. An idea of an immunity passport has been mooted and it involves the certification of COVID-19 survivors [71]. If the reports of possible re-infection within a year have been disproved then individuals with an ‘immunity passport’ could be recruited as volunteer workers to assist at the hospitals or isolation centers. Nutrition and lifestyle: antioxidation combat oxidative stress; builds the immune system and reduces vulnerability to the virulence of SARS-CoV-2. The daily consumption of plenty of fruits and vegetables ensures that more antioxidants are made available within our biological systems. To ensure adequate consumption of fruits and vegetables, antioxidant beverages can be made out of them. Indeed, these beverages can be made from the water extracts of any edible plant whose acute toxicity dose is not less than 5,000mg/kg [72,73].

Apart from the prophylactic benefits, the aggressive consumption of antioxidant beverages has potential therapeutic value in the face of a COVID-19 infection. This is because these drinks usually have strong anti-inflammatory properties. Adequate vitamin C supplementation is also good for the management of COVID-19 due to their antioxidant and anti-inflammatory effect. It has also been shown to stimulate the production of interferon type 1 which SARS-CoV-2 suppresses [74]. Clinicians should strive to determine the maximum beneficial dose as research shows that many COVID-19 patients might be under-dosed with vitamin C. Heavy consumption of refined sugar should be avoided as its oxidative effect impairs immune function [18]. Chronic exposure to tobacco smoke and air pollution should be avoided as these induce pulmonary stress and weakens the ability of the lungs to fight coronavirus infection. Individuals should engage in constant exercise as it helps to increase the lung volume and it is a form of antioxidation [75]. Heavy alcohol consumption weakens the immune system and should be avoided [76]. Management of COVID-19 patients: on admission of COVID-19 patients, an early determination of the Neutrophil to Lymphocyte Ratio (NLR) would help to determine the degree of severity of cases [64]. This would help in the dedication of more attention to the would-be severe cases. The administration chloroquine (CQ) and hydroxychloroquine (HCQ) to patients is encouraged as this has positively imparted on the clinical outcomes in severe cases. This is because these drugs have both antiviral and immunomodulatory properties [77,78]. However, there is an urgent need to discover (or design) and develop specific novel drugs against SARS-CoV-2 infection, especially as none exists currently and the number of infected individuals and mortality continues to rise globally.

Medicinal plants are used a lot in folkloric medicine for treatment of various ailments including viral diseases. Therefore, we recommend extensive investigation of medicinal plants with antiviral potential in order to identify novel therapeutic molecules that may elicit strong inhibitory effects on various SARS-CoV-2 target proteins. The bioactive ingredients of these plants should have high efficacy and little or no adverse effects on the patient. More so, medicinal plants with anti-inflammatory properties may also be explored as they may contain natural compounds that can help mitigate the cytokine storm and its effects that form part of SARS-CoV-2 pathology. In this regard, the herbal drink, COVID-organics claimed to combat COVID-19 in Madagascar should be investigated. Research and testing: the role of NK cells in combating COVID-19 should be investigated since these are the cells responsible for immunosurveillance of virally infected cells [79]. Immunological data from individuals with severe cases should be obtained. Information on the genetic susceptibility to COVID-19 can be obtained from extensive HLA profiling of patients especially when the severity of the case cannot be associated with any known risk factor [53]. Biomedical researchers should develop cheap kits using surrogate markers for the mass testing of the immune status of individuals. This would provide individuals the basic information of their readiness to combat a possible infection. As more animal carriers are been discovered, the zoonotic transmission of SARS-CoV-2 should be critically studied vis-à-vis infectivity and rates of mutations in these hosts. Tracking of the SARS-CoV-2 genome for mutations would help to provide data for researchers to understand its virulence and make future projections. Consequently, scientists would be able to genetically predict the next SARS-CoV strains and develop strategies to prevent the re-emergence of another pandemic [80]. A summary of these recommendations is provided in Table 1.

**Table 1:** recommendations for COVID-19 stakeholders

Stakeholder	Suggested Activity
National Government	Mass orientation on immune enhancement as a way of combating COVID-19
Researchers	Vaccine development using the immunogenic peptides of SARS-CoV-2 conserved regions
	Develop vaccines and antivirals for domesticated animals
	Extensive serological survey for post-COVID-19 patients.
	Investigate the role of Natural Killer cells in combating COVID-19
	More studies on the zoonotic transmission of SARS-CoV-2
	HLA profiling for testing genetic susceptibility
	Develop cheap kits for the mass testing of the immune status of individuals.
	Track SARS-CoV-2 genome mutations.
	Discovery of SARS-CoV-2 inhibitors from natural products
	Investigate the safety and therapeutic potential of Madagascar’s COVID-19 herbal drink
Clinicians	Early determination of case severity through the Neutrophil to Lymphocyte Ratio
	Admin. of safe antioxidant beverages to reduce hospitalization time
	Admin. of CQ or HCQ to patients due to their antiviral and immunomodulatory properties
Non-Infected citizens	Aggressive antioxidation through fruits and vegetables
	Reduce consumption of refined sugar
	Reduce exposure to tobacco smoke and air pollution
	Reduce consumption of alcohol
	Regular physical exercises
	Determine immune status through test kits
COVID-19 survivors	Assist as volunteers in hospitals and isolation centers

## Conclusion

The world is facing a daunting challenge of a COVID-19 pandemic ravaging the health and economies of its nations and territories. As the world prospects for a cure, there is a salient answer in the immune system which can be fully discovered, explored and optimized. In the control of the pandemic, it is proper to give equal or even more attention to the immune system which provides an approximately 95% cover to infected persons. Beyond doubt, the prevention, cure and management of COVID-19 is a dynamic interplay between viral inhibition, cellular antioxidation and immunomodulation. It is therefore necessary to ensure good nutrition, a healthy lifestyle, a functional antioxidant defense system and balanced immune system to prevent the exacerbation of the disease. While the world awaits the emergence of an effective vaccine, another proactive strategy (without violating the WHO standard preventive measures) is to prepare the citizenry for a possible invasion of the ‘invisible enemy’ by other immune enhancement measures. Through mass orientation, people should be taught that their immune system is a project which needs to be properly nurtured to accurately recognize, swiftly respond and protractedly remember SARS-CoV-2 infections and indeed other pathogenic threats.
